# Integrating genome annotation and QTL position to identify candidate genes for productivity, architecture and water-use efficiency in *Populus* spp

**DOI:** 10.1186/1471-2229-12-173

**Published:** 2012-09-26

**Authors:** Romain Monclus, Jean-Charles Leplé, Catherine Bastien, Pierre-François Bert, Marc Villar, Nicolas Marron, Franck Brignolas, Véronique Jorge

**Affiliations:** 1 INRA, UR0588 Amélioration Génétique et Physiologie Forestières (AGPF), F-45075, Orléans, France; 2 UFR-Faculté des Sciences, UPRES EA 1207 Laboratoire de Biologie des Ligneux et des Grandes Cultures (LBLGC), Université d'Orléans, F-45067, Orléans, France; 3 INRA, USC1328 Arbres et Réponses aux Contraintes Hydriques et Environnementales (ARCHE), F-45067, Orléans, France; 4 INRA, UMR1137 Écologie et Écophysiologie Forestières (EEF), F-54280, Champenoux, France; 5 Université de Lorraine, UMR 1137, Ecologie et Ecophysiologie Forestières (EEF), Faculté des Sciences, F-54500, Vandœuvre-lès-Nancy, France; 6 Present address: INRA, UMR1287 Ecophysiologie et Génomique Fonctionnelle de la Vigne, F-33882, Villenave d'Ornon, France

## Abstract

**Background:**

Hybrid poplars species are candidates for biomass production but breeding efforts are needed to combine productivity and water use efficiency in improved cultivars. The understanding of the genetic architecture of growth in poplar by a Quantitative Trait Loci (QTL) approach can help us to elucidate the molecular basis of such integrative traits but identifying candidate genes underlying these QTLs remains difficult. Nevertheless, the increase of genomic information together with the accessibility to a reference genome sequence (*Populus trichocarpa* Nisqually-1) allow to bridge QTL information on genetic maps and physical location of candidate genes on the genome. The objective of the study is to identify QTLs controlling productivity, architecture and leaf traits in a *P. deltoides* x *P. trichocarpa* F1 progeny and to identify candidate genes underlying QTLs based on the anchoring of genetic maps on the genome and the gene ontology information linked to genome annotation. The strategy to explore genome annotation was to use Gene Ontology enrichment tools to test if some functional categories are statistically over-represented in QTL regions.

**Results:**

Four leaf traits and 7 growth traits were measured on 330 F1 *P. deltoides* x *P. trichocarpa* progeny. A total of 77 QTLs controlling 11 traits were identified explaining from 1.8 to 17.2% of the variation of traits. For 58 QTLs, confidence intervals could be projected on the genome. An extended functional annotation was built based on data retrieved from the plant genome database Phytozome and from an inference of function using homology between *Populus* and the model plant *Arabidopsis*. Genes located within QTL confidence intervals were retrieved and enrichments in gene ontology (GO) terms were determined using different methods. Significant enrichments were found for all traits. Particularly relevant biological processes GO terms were identified for QTLs controlling number of sylleptic branches: intervals were enriched in GO terms of biological process like ‘ripening’ and ‘adventitious roots development’.

**Conclusion:**

Beyond the simple identification of QTLs, this study is the first to use a global approach of GO terms enrichment analysis to fully explore gene function under QTLs confidence intervals in plants. This global approach may lead to identification of new candidate genes for traits of interest.

## Background

Dissection of genetic architecture of complex trait such as growth and yield in plants has been achieved by Quantitative Trait Loci (QTL) detection approaches. Dissecting loci to find the causative genes can be considered as the Holy Grail for geneticists. The ultimate road to find the genes, positional cloning, has been achieved in main crop plants [1] but it is slow and labour intensive especially because large segregating populations have to be developed. The advent of plant whole genome sequences has opened the possibility of anchoring genetic maps and positioning QTL on a physical map. Nevertheless, QTL intervals correspond to several hundreds of genes [1]. Despite of some successful positional cloning stories in plants, there is room for complementary approaches, like association mapping (reviewed in [2]) or integrative –omics strategies [3], proposed to narrow intervals and length of candidate gene lists to be studied further [1].

The identification of Quantitative Trait Loci (QTL) has already been reported for poplar species including several yield and growth components such as stem dimension (height, circumference) and architecture [4-16]. These studies together identified more than 600 QTLs explaining up to 73% of the trait variation, with confidence intervals ranging from 2.1 to 261.2 cM. These global summary statistics confirm the common idea that growth traits in *Populus* are controlled by many loci. However, ranges of variation explained and size of confidence intervals highly depend on the mapping population used and the environment(s) in which traits are measured.

Today, the anchoring of genetic maps and QTLs on *Populus trichocarpa* genome sequence [17] allows identifying large genome regions containing several hundred of genes. In order to reduce this number to a reasonable number of candidate genes, one strategy consists in selecting candidate genes based on functional knowledge (for ex. known biosynthetic pathways; transcriptomic data; annotation inferred from homologous genes in other species) and check if the candidates co-locate with QTLs [11,18,19]. Another strategy, applied in *Populus* spp., consists in combining QTL position, structural information and transcriptomic experiments to refine a gene list for functional characterization [11,20]. The present study focused on traits that are of notable importance for poplars biomass production systems, where juvenile growth, architecture and water use efficiency (WUE) are the main criteria for breeding programs. Among published QTL studies carried out on these traits, few have anchored QTLs on the poplar genome to identify and analyse the underlying large candidate gene lists [9,11]. Today, the biological interpretation of these gene lists is made possible by the availability of biological knowledge accumulated in public databases (e.g. Gene Ontology) and bioinformatic high-throughput enrichment tools [21]. In this study, Ontologizer [22] was used in order to analyse gene sets included in QTL confidence intervals and we tested if QTL regions are statistically enriched in some functional categories compared to the entire genome.

The objectives of this study were: (1) to identify QTLs controlling productivity, architecture and leaf traits in hybrid poplars; (2) to identify candidate genes under QTL intervals using enrichment tools and Gene Ontology.

## Results

### Trait variation, distribution and relationship between traits

Frequency distributions were not significantly departed from normal distribution (data not shown; Additional file 1). Genotype effect was significant for all traits (Data not shown). The *P. deltoides* parent showed higher overall growth in height and circumference than the *P. trichocarpa* parent (Table 1). The progeny showed generally a higher growth than both parents. A proportion of transgressive segregation (considered as heterosis by other authors) was calculated and it was high for all traits related to growth which was not the case for leaf traits. The coefficients of genetic variation (CV_G_) ranged from 0.7 to 20.3%. Values of heritability at genotype level were moderate to high (*H*^*2*^_Genotype_: 0.32 to 0.72) and *H*^*2*^_Individual_ was low to moderate, ranging from 0.09 to 0.31.

**Table 1:** **Parental means, progeny mean,genotypic range, coefficient ofgenetic variation and heritabilitiesfor each trait measured**

** Trait**	***P. deltoides *73028**–**62 mean**	***P. trichocarpa *101**–**74 mean**^**a**^	**Progeny mean ± SE**	**Genotypic range**^**c**^	**% of transgressive segregation**	**CV**_**G**_**%**	***H***^***2***^_***Ind***_ ^**b**^	***H***^***2***^_***Gen***_ ^**b**^
Circum1 (mm)	48.0	21.1	46.03 ± 0.31	32.1-64.4	33.2	9.0	0.16	0.53
Height1 (cm)	253.5	185.7	286.29 ± 1.32	224.8-358.3	30.4	6.5	0.26	0.53
Circum2 (mm)	114.3	56.3	118.80 ± 0.62	82.9-150.7	39.3	16.3	0.14	0.50
Height2 (cm)	512.8	387.3	600.10 ± 2.37	473.5-724.7	33.3	5.5	0.19	0.58
Syllep1	14.4	16.8	21.17 ± 0.28	8.3-35.7	35.7	20.3	0.31	0.72
deltaC (mm)	66.3	35.2	73.95 ± 0.40	50.1-96.6	45.7	7.4	0.18	0.57
deltaH (cm)	259.3	201.6	314.90 ± 1.73	226.4-396.5	36.6	13.4	0.28	0.69
SLA (cm^2^.g^-1^)	121.8	153.7	142.84 ± 0.46	119.1-165.6	3.7	4.4	0.22	0.58
*C*_M_ (mg.g^-1^)	443.0	453.5	454.34 ± 0.26	437.2-469.7	1.36	0.7	0.14	0.45
*N*_M_ (mg.g^-1^)	20.3	14.8	20.46 ± 0.11	14.4-26.2	16.5	7.8	0.26	0.63
Δ ( ‰)	21.2	21.2	21.60 ± 0.02	20.2-22.9	1.89	1.1	0.09	0.32

^a^ based on two ramets only.

^b^ Individual (Ind) and genotypic (Gen) heritabilities.

^c^ The minimum and the maximum of genotypic means.

All leaf traits were significantly correlated, either negatively or positively and the highest correlation coefficients were observed between SLA and Δ (0.37), and between SLA and N_M_ (0.36; Additional file 1). All productivity traits were significantly correlated except syllep1 with deltaH. The strongest correlations were observed between height and circumference within and between years, and between annual growth (deltaH, deltaC) and the second year measurements (height2, circum2). Carbon isotope discrimination is negatively correlated with all productivity traits. Number of sylleptic branches was correlated more tightly with circumference than with height. Correlations between leaf and productivity traits of the first year were higher than with second year productivity traits.

### Genetic maps and QTL analysis

**Figure 1:**
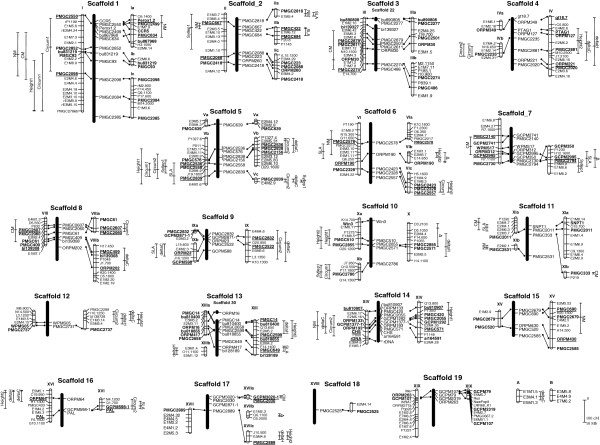
**Framework linkage maps andQTLs from the segregationanalysis of the *P. deltoides *x *P. trichocarpa *pedigree aligned on *P. trichocarpa *Nisqually-1 sequence.** Genome version assembly was Phytozome annotation v2.2. From the left to the right, *P. deltoides* female 73028–62 genetic map (in white), *P. trichocarpa* Nisqually-1 physical map with position of genome anchored markers (in black), and *P. trichocarpa* 101–74 male genetic map (in white). Scaffolds were numbered according to the v2.2 genome version. Additionally, extra scaffold (>19) containing markers mapped on the genetic maps were also shown. The length of bars is proportional to the map distance in cM or to sequence length in bp. Marker names are explained in Material and Methods. Markers in bolds are anchoring markers. Markers in bold and underlined are QTL flanking markers used for the projection of QTL confidence intervals on the physical map. QTLs were represented by vertical lines with horizontal small lines indicating start and stop of the confidence intervals and position of the LOD peak. Trait names were explained in Material and Methods.

Among the 110 new SSR tested, only 6 were distorted and, as they were not linked between them, they were discarded for the linkage analysis. Genetic maps used for the detection of QTLs and their projection on the genome are summarized in Additional file 2. Briefly, genetic maps cover 3 126 cM and 3 222 cM for the *P. deltoides* and *P. trichocarpa* maps respectively, with a mean distance between markers of 18.75 cM. The mean numbers of marker per linkage groups were 8.0 and 6.1 for *P. deltoides* and *P. trichocarpa* respectively. A total of 67 and 81 markers anchored to the genome sequence for *P. deltoides* and *P. trichocarpa* respectively. A mean of 3.5 and 4.2 markers were anchored per linkage groups. All of them, with the exception of 2 linkage groups of the *P. trichocarpa* genetic map, were assigned to a chromosome (Figure 1). Estimated genome coverage was 78% and 66% for *P. deltoides* and *P. trichocarpa* map respectively.

The results of QTL detection are listed in Additional file 3 and details on genetic maps and QTL positions are graphically presented in Figure 1. A total of 77 QTL were detected explaining between 1.8% and 17.2% of the trait variation (at chromosome level *P-*value threshold of 0.05). The maximum was reached for N_M_ on linkage group II on the *P. deltoides* map. An average of 3.3 and 3.7 QTLs were detected per trait respectively on the *P. deltoides* and *P. trichocarpa* maps, and total explained variance varied from 4.9% to 34.7%, maximum being reached by N_M_. Thirty five QTLs were detected on the *P. deltoides* map and 42 on the *P. trichocarpa* map.

All QTLs were not distributed evenly on the genome (*P*-value of chi squared test: 7 10^-9^), and one linkage group (V) came out to be a hot spot with 10 QTLs. Among the 27 QTLs controlling height and circumference measured the first and the second year, only few cases of co-location were identified (LG IV, LG V and LG X).

### QTL projection on the genome

**Figure 2:**
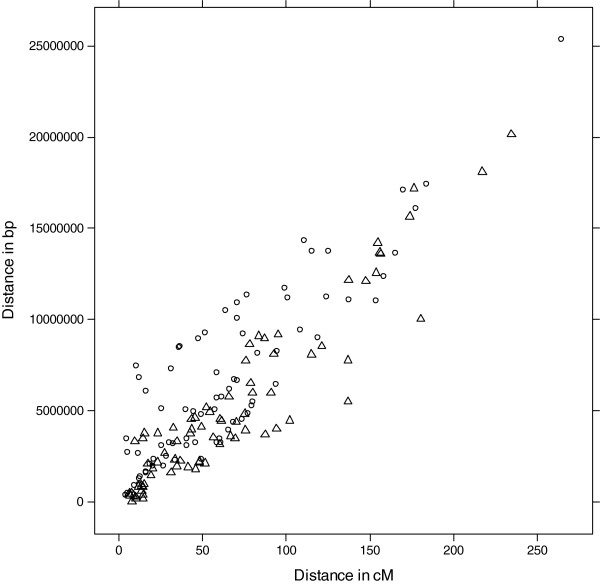
**Global relationship between physicaldistance (in bp) andgenetic distance (in cM).** The relationship is shown for both parental maps, *P. deltoides* map (circle), *P. trichocarpa* (triangles). Each point represents a physical and a genetic interval between 2 markers within a chromosome/linkage group.

Among the 126 markers having sequence information (primer and gene sequences), only 10 did not show matching or showed inconsistent matching on the genome sequence (data not shown). Genetic and physical positions for 116 markers were used to calculate and compare within chromosome pair wise genetic and physical distances (Figure 2; Additional file 4). The global ratio between physical distance (bp) and genetic distance (cM) was 95,184 bp/cM for the *P. deltoides* map and 77,803 bp/cM for the *P. trichocarpa* map. This ratio varied between linkage groups, from 42,413 to 132,309 bp/cM for the *P. deltoides* linkage groups, and from 9,487 to 230,033 cM for the *P. trichocarpa* linkage groups.

Among the 77 detected QTLs, only 19 could not be projected on the genome (Additional file 3). For 18 QTLs, the corresponding LG carried only one marker that could be anchored on the genome and this configuration did not allow to correctly orientate the QTL on the genome. For one QTL (LG A), there was no anchoring marker. Mean size of projected QTL interval was 11,099,561 bp for *P. deltoides* and 8,096,353 bp for *P. trichocarpa*. Mean number of genes included in all QTLs confidence intervals for a particular trait was 4,216 and varied between 2,445 (Syllep1) and 5,937 (Height1; Table 2).

**Table 2:** **Global analysis of geneannotation and gene ontology(GO) among gene setby traits**

**Trait**	**Number of gene GOannotated / total numberin the set**^**a**^	**Percentage of annotated genes**^**b**^	**Numbers of GO termsanalyzed**	**Number of GO significantlyover-represented**^**c**^	**Percentage of significant GOterms identified**^**d**^
Height1	5217 / 5937	87.87	4323	34	0.78
Height2	3445 / 3882	88.74	3657	66	1.80
Circum1	4601 / 5276	87.20	4255	36	0.84
Circum2	3054 / 3457	88.34	3631	48	1.32
deltaH	2552 / 2856	89.35	3232	45	1.39
deltaC	4422 / 4990	88.61	4025	70	1.70
Syllep1	2168 / 2445	88.67	2993	124	4.14
SLA	3272 / 3712	88.14	3666	98	2.67
N_M_	4943 / 5609	87.56	4263	37	0.86
C_M_	4747 / 5405	87.82	4149	75	1.80
Δ	2450 / 2775	88.28	3363	51	1.51

^a^ Size of the total population is 40,668 genes and 35,467 have at least one correspondence with a GO term.

^b^ The mean is 88.23 ± 1.18 (95 % confidence interval).

^c^ Topology-Weighted analysis and p-value < 1 %.

^d^ The mean is 1.71 ± 1.90 (95 % confidence interval).

### GO terms enrichment in QTL confidence intervals

Projecting the QTLs into physical genomic regions provided the opportunity to search for possible enrichment in gene functions that could be related to the traits under study. A rational and without *a priori* strategy to do this is to analyse the Gene Ontology (GO) annotation associated with the gene sets. The available Gene ontology (GO) annotation was relatively limited for *Populus trichocarpa* as compared to the model plant *Arabidopsis thaliana*. Indeed, searching for GO association in Phytozome plant genome database, 18,542 gene models have been found associated with at least one GO term (data not shown). In order to increase the number of annotated gene models, a *Populus* GO annotation has been inferred based on protein-protein similarity between *Arabidopsis* and poplar. At least one GO term annotation could be retrieved for 35,467 genes among the 40,668 poplar gene models identified in Phytozome, which represent 87.21% of genes annotated (see Material and Methods). Main results obtained from the gene ontology analysis are presented in Table 2. For each QTL intervals, the percentage of annotated genes did not differ significantly from the 87.21% determined above. Consequently, enrichment analysis was not biased by a distortion in the percentage of annotated genes within the QTLs. At a *P*-value threshold of 1%, the number of over-represented GO terms varied between 34 and 124 depending on the considered trait. The percentage of significantly over-represented (enriched) GO terms on total number of GO terms for a particular trait varied between 0.78% and 4.14%. There was no significant correlation between the cumulated size of QTL confidence intervals on the genome for a particular trait and the number of significant GO terms in the corresponding intervals (*P-*value = 0.21). Remarkably, a significant higher percentage of over-represented GO terms was observed for the gene set included in confidence intervals of all QTLs controlling the number of sylleptic branches (Syllep1). Interestingly, this trait showed the highest heritability (*H*^*2*^_Individual_ and *H*^*2*^_Genotype_) and coefficient of genetic variation (CV_G_). Thus, we decided to explore further gene ontology enrichment within QTLs controlling number of sylleptic branches (Syllep1). Complete lists of GO terms significantly enriched are presented in Additional file 5.

### Functional analysis of genes in QTL confidence intervals controlling number of sylleptic branches

**Figure 3:**
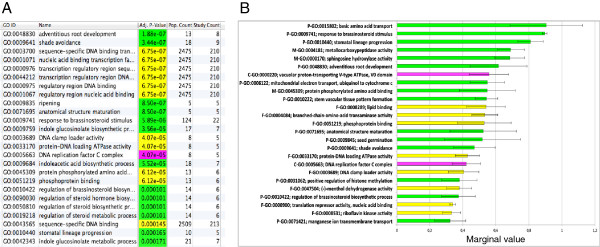
**Comparison of enrichment analysesof GO terms usingTW or MGAS methods.** Application to genes within QTLs for the number of sylleptic branches. (**A**) Ranked list of the 25 overrepresented terms using a Topology-Weighted (TW). (**B**) Ranked list of the top 25 terms identified by 20 runs of MGSA. Error bars (95 % confidence intervals) obtained with the 20 runs of MGSA. In case of MGSA each of the 25 terms was identified with a marginal value >0.5 in at least one of the 20 runs. GO:xxxxxxx: gene ontology accession; Green label and “P” prefix to GO accession refer to the ontology domain “biological process”; Yellow label and “F” : “molecular function”; magenta label and “C”: “cellular component”.

An additional enrichment method called MGSA for model-gene based analysis, developed recently and integrated to the Ontologizer webtool, has been tested. This method is presented as faster and more accurate in identifying less redundant GO terms than previous methods such as the Topology-weighted methods [23]. Figure 3A presents a detailed view of the enrichment analysis using the Topology-Weighted (TW) method. Only one run is needed with this method that provides a *P*-value associated with each GO term. As already mentioned earlier by [23], the TW method may give some redundant results as observed for example in lines 3 to 8 for the GO term related to DNA binding. Results were not so redundant with MGSA (Figure 3B). However, the main disadvantage of MGSA is the lack of consistency between replicated analyses. This could be due to the Bayesian approach and must be taken in consideration. Several runs of MGSA were performed in order to be able to have a ranking of the most pertinent GO terms and to compare this ranking between TW and MGSA (Figure 3, Table 3). Significant variability between the 20 runs of MGSA was observed leading to the conclusion that the MGSA method did not gave reliable results. However, when comparing the first 10 terms identified using both enrichment methods, common GO term were found corresponding to the biological processes of adventitious root development (Table 3). The second most significant GO term referred to the process of shade avoidance, which could make sense regarding branch formation. If the 210 DNA binding and transcription factors terms identified were removed considering them as generic and thus not so biologically informative, the third enrichment class corresponded to genes potentially involved in the ripening process, an important process in fruit development but somewhat intriguing in shoot development. These three sets were analysed further.

**Table 3:** **The first ten rankedGO terms represented withinthe QTLs controlling numberof sylleptic branches**

** GO ID**	**Name**	**P-value**^**a**^	**Count**^**b**^	**Ontology**^**c**^
**Model-based gene set analysis**				
GO:0015802	Basic amino acid transport	0.9050	9 / 26	P
GO:0009741	Response to brassinosteroid stimulus	0.8964	22 / 124	P
GO:0010440	Stomatal lineage progression	0.8092	5 / 10	P
GO:0004181	Metallocarboxypeptidase activity	0.6898	3 / 4	F
GO:0000170	Sphingosine hydroxylase activity	0.6842	3 / 3	F
GO:0048830	Adventitious root development	0.61586	8 / 13	P
GO:0000220	Vacuolar proton-transporting V-type ATPase. V0 domain	0.5584	3 / 4	C
GO:0006122	Mitochondrial electron transport. ubiquinol to cytochrome c	0.5481	4 / 8	P
GO:0045309	Protein phosphorylated amino acid binding	0.5478	6 / 13	F
GO:0010222	Stem vascular tissue pattern formation	0.5430	3 / 5	P
**Topology-Weighted**				
GO:0048830	Adventitious root development	1.8770 e-07	8 / 13	P
GO:0009641	Shade avoidance	3.4385 e-07	9 / 18	P
GO:0003700	Sequence-specific DNA binding transcription factor activity	6.7533 e-07	210 / 2475	M
GO:0001071	Nucleic acid binding transcription factor activity	6.7533 e-07	210 / 2475	M
GO:0000976	Transcription regulatory region sequence-specific DNA binding	6.7533 e-07	210 / 2475	M
GO:0044212	Transcription regulatory region DNA binding	6.7533 e-07	210 / 2475	M
GO:0000975	Regulatory region DNA binding	6.7533 e-07	210 / 2475	M
GO:0001067	Regulatory region nucleic acid binding	6.7533 e-07	210 / 2475	M
GO:0009835	Ripening	8.4975 e-07	5 / 5	P
GO:0071695	Anatomical structure maturation	8.4975 e-07	5 / 5	P

^a^ The column ‘p-value’ indicates 1) for MGSA: the marginal value of a term being in the ‘active’ state; thus a high value gives a strong evidence for an association; the mean marginal value from 20 runs is indicated (see also Figure 3); 2) for TW: the probability of observing at least the same amount of enrichment when significant genes are randomly selected out of all genes; thus, a very small value gives strong evidence for an association.

^b^ the counts of genes in the study (x) and population (y) sets as x / y.

^c^ P, F, C refer to the three classes of the gene ontology: biological process, molecular function and cellular component respectively.

Topology-Weighted (TW) and Model-based Gene Set Analysis (MGSA) results are shown.

Eight genes corresponding to adventitious root development were found: POPTR_0002s02690, POPTR_0002s02700, POPTR_0002s02730, POPTR_0002s02740, POPTR_0002s02770, POPTR_0002s02790, POPTR_0002s02800, POPTR_0002s09050 (Additional file 6). All these genes belonged to the chromosome 2. In the text below, all gene names refer to *Arabidopsis* naming nomenclature. Annotation was analysed looking to the peptide homologs on Phytozome (See Additional file 6). Seven genes encode cytochrome P450, family 83, subfamily B and their protein homologs in *Arabidopsis* correspond to *CYP83B1* / *SUR2*. The percentage of similarity at the amino acid level was between 60% and 65%. The last gene, POPTR_0002s09050, encodes an *ARF17* homolog. These 8 genes were also gathered under the enriched GO term related to shade avoidance identified as the second most significant term. In addition, two other genes were within this group: POPTR_0002s06090 and POPTR_0005s22330. They were localized on chromosome 2 and chromosome 5 and they encode *PAR2* (AT3G58850) homologs.

The third selected enrichment gathered five genes related to the biological process of ripening as inferred from ontology annotation in *Arabidopsis* (POPTR_0002s10240. POPTR_0002s10250. POPTR_0002s13340. POPTR_0005s17480. POPTR_0007s14390; Additional file 6). The official GO annotation of the two *Arabidopsis* homologous transcripts identified here (AT5G65380.1 and AT1G47530.1) was inferred from sequence similarity with a ripening regulated protein *DDTFR18* identified in *Solanum lycopersicum* (GenBank accession AAG49032.1). The five poplar homologues genes originated from three different chromosomes. They encoded proteins belonging to the multidrug and toxic compound extrusion (MATE) protein family, included in the large superfamily of multidrug transporters [24].

## Discussion

### Are trait variation and correlations among traits consistent with previous studies?

The baseline for all genetic improvement is the availability of information on genetic variation for the traits of interest. Efforts have been made to describe growth and WUE variations and relationships among traits in different unrelated poplar cultivars and hybrid families under different water regimes [25-32]. High levels of genotypic variability for productivity and for Δ have been found under non limited water supply or moderate water deficit. In the present study, progeny mean values for stem height at the end of the first and second growing seasons were in agreement with those previously reported for other *P. deltoides* x *P. trichocarpa* F1 large family on the same trial [5,33]. Nevertheless, the range of values was lower. In a subset of 33 genotypes of the same family measured in an adjacent trial [29], height and circumference means were not comparable because of the trial management (pruned each year), but for leaf traits, mean values were similar, showing that the trial management did not influenced leaf trait variability. On the contrary, for a different *P. deltoides* x *P. trichocarpa* F1 [34] measured on the same trial, parental and progeny means for leaf traits (SLA, N_M_, C_M_), were higher than those reported in the present study.

A proportion of transgressive segregation was calculated to make comparisons with previous studies where it was considered as heterosis [5]. This proportion was lower for Circum2, deltaC and deltaH. These differences could be explained by a difference in circumference for the *P. trichocarpa* parent. The *P. trichocarpa* 101–74 parent of the present study had a lower juvenile growth than the *P. trichocarpa* ‘V24’ parent from [5]. For *Populus* spp., parental specific effect can explain a significant part of the trait variation in progenies (Bastien et al., in prep.). For leaf traits, low or no significant levels of transgressive segregation were observed as previously reported [5].

Analysis of the relationship among traits measured the first year shows that productive clones (high circumference) were characterized by low SLA (dense/ thick leaves) and low carbon isotope discrimination (high water-use efficiency). Water use efficient clones (low Δ) were characterized by low SLA (dense/thick leaves) and low leaf nitrogen content. Very low or no correlation was observed between leaf nitrogen content and growth traits, which is contradictory with previous studies on the subset of the same progeny or other progeny [29,35]. The strength of the links between productivity and Δ differ according to poplar species: a positive relationship was found for *Populus davidiana*[25], whereas negative correlations were detected for *P. trichocarpa* × *P. deltoides* and no correlation was observed in *P. deltoides* × *P. nigra*[26-28,36]. Occurrence of genetic variability and no systematic trade-off between these traits suggest that it should be possible to select genotypes combining large levels of productivity and WUE. The different trial managements and genetic or species backgrounds could explain these discrepancies.

### Could QTLs be identified for growth and leaf traits?

Given the range of variation, the significant genotype effect and levels of heritability for all traits measured, significant results from the QTL detection were expected. Average QTL number for each trait was very similar to those reported previously [5] but less than average QTL number reported elsewhere [8,9]. This could be explained by a larger trait variation existing in a F2 progeny in the latter studies. A hot spot of QTL co-location on LG V was related to growth traits and SLA. These results were in agreement with the negative correlation observed between both types of traits. Additionally, the opposite effects of QTLs for both types of traits were also in agreement with the sign of trait correlation (LG Vb from *P. deltoides* map; Additional file 3). This linkage group in this particular pedigree was also involved in bud phenology (explaining up to 25% of the budset variation, [18]). Growing season length explained a large part of productivity in poplar [34] and could explain the colocation of growth and budset QTLs. On the other hand, for highly correlated traits, like circumference and height measured in the first and second growing seasons, the observed number of co-locations was lower than expected. This could be partly explained by the cut off (*P-*value) for declaring a QTL, which eliminate QTLs that were just below the threshold (data not shown). Additionally, the high correlation might be driven by plant intrinsic mechanistic correlations between traits.

All QTLs controlling Δ were on the *P. trichocarpa* parental map. Despite of a significant negative correlation between circumference measured the first year (Circum1) and carbon isotope discrimination (Δ), no QTLs co-location (overlapping confidence intervals) has been detected for these two traits. The map coverage was not exhaustive and could explain the absence of co-location. Nevertheless, availability of unlinked QTLs for different trait of interest has a particular advantage for breeding purposes: it opens here the possibility to select clones combining both high productivity and high water efficiency.

For comparison with published QTLs in other mapping pedigrees, we focused on studies with the same *Populus* species and with genetic maps carrying a significant number of genome anchoring markers [5,8,9]. In many cases, co-locations occurred on the same chromosomes only when pairs of studies were compared (Additional file 7). In only one case (LG X), a co-location occurred in the four studies compared ( [5,8,9] and the present study; Additional file 8). There were very few common anchoring markers between all genetic maps, which impeded a meta-analysis of QTLs, but anchoring on genome allowed aligning all the maps. The genome interval to consider included actually almost the whole chromosome, and need to be narrowed for further analysis and interpretation.

### Could candidate genes for leaf and growth traits be identified through GO term enrichment analysis within QTL confidence intervals?

It was the first time GO term enrichment analysis is used in plants to identify putative candidate genes for QTLs. Generally, *a priori* identified candidate genes were searched within QTL intervals: their presence therefore “validate” putative important gene function related to the trait (see for example [37-39]). However, a recent analysis in animals provided evidence of over represented GO terms in QTL regions of the bovine genome [40]. Moreover, the authors showed that enrichment classifications are consistent with the trait category controlled by the QTLs. In the present study, for all traits, significant GO terms enrichment were found in QTL confidence intervals. For QTLs controlling sylleptic branches, 8 genes linked to the GO: 0048830 defining the biological process of adventitious root formation were found. These genes not only belong to the chromosome 2 but they are also clustered together on the chromosome. So they may have been identified by the enrichment methods because they are duplicated in tandem and not because they could be involved in the trait variation. This remark is also true for all associations found. However, the significant association due to a physical linkage between genes seems less plausible when the significant GO terms are associated with genes from different chromosomes. Five genes identified correspond to the enrichment in GO term related to ripening. These 5 genes are located on 3 different chromosomes. In that case, the association due to tandem duplication biases of this enrichment seems unlikely.

### What is the meaning of finding enrichment in biological processes such as adventitious root formation and ripening when studying sylleptic branches formation?

In perennial dicotyledonous species such as poplar, sylleptic branches are formed either on the elongating stem during the growing season or at the time of regrowth, from arrested axillary buds formed the year before. In the present study, the number of sylleptic branches was measured at the end of the first growing season and do not proceed from axillary bud break. Therefore, sylleptic branches formation measured here might have some common features with the mechanism of shoot branching well studied in model plant such as *Arabidopsis*. The two class of genes related to adventitious root formation were *SUR2* and *ARF17* homologs. These GO classification make sense because *SUR2* defines the first step in making indolic glucosinolates from indole-3-acetaldoxime (IAOx). Loss-of-function *sur2* mutants block the production of glucosinolates from IAOx, leading to an increased IAOx flux for IAA biosynthesis. In that way, *SUR2* participates in auxin homeostasis [41,42]. Likewise, *ARF17* (AT1G77850) belongs to the *AUXIN RESPONSE FACTOR* (*ARF*) gene family products which together with the AUXIN / INDOLE-3-ACETIC ACID (Aux/IAA) proteins regulate auxin-mediated transcriptional activation / repression [43]. *ARF17* was predicted to control adventitious rooting by modulating indole-3-acetic acid (IAA) homoeostasis [44]. Several hormones such as auxin, cytokinin, and the newly identified strigolactones are known to act directly or indirectly on bud outgrowth and to control shoot branching [45-48]. Consequently, the identified poplar genes could be related to auxin homeostasis regulation in shoot branching rather than adventitious root formation; and this makes sense with respect to the trait under study. These 8 genes were also gathered under the enriched GO term related to shade avoidance identified using the Topology-Weighted (Table 3; Additional file 6). In addition, two other genes are within this group: POPTR_0002s06090 and POPTR_0005s22330. They are localized on chromosome 2 and chromosome 5 and they encode *PAR2* (AT3G58850) homologs. *PAR2* functions as transcriptional repressor of auxin-responsive genes *SAUR15* (AT4G38850) and *SAUR68* (AT1G29510). It is obvious that the similarity was very low and thus the function largely undetermined. However, their co-localization with the *SUR2* and *ARF17* homologs was an argument to explore further their function in auxin response signalling in poplar.

### Are the MATE-type transporters involved in auxin/strigolactones signalling?

The second most significant enrichment gathered five genes related to a GO term defining ‘ripening’. These five genes encode putative MATE efflux family proteins. MATE proteins are believed to function as proton-dependent efflux transporters. These classes of proteins were identified through their trans-membrane protein domain and were grouped in pfam database [49] under the pfam family accession PF01554. In Phytozome database, 73 poplar proteins carrying this protein domain were found. Similarly, 56 putative MATE proteins in *Arabidopsis* were identified. This is consistent with previous studies indicating that MATE proteins have exceedingly large numbers of homologs in plants per species, in that aspect contrasting to what is observed in bacteria and animals [50,51]. But little is known about their function in plants. One can hypothesize that the genes identified here could have a function in shoot branching through hormones regulation. In mammals, MATE-type transporters recognize hydrophobic hormones and transmitters, such as testosterone and corticosterone [50]. In *Arabidopsis*, *TT12* was a MATE efflux protein proposed to transport glycosylated flavan-3-ols *in vivo*[52]. Therefore, plant MATE efflux proteins represent interesting short/long-distance transporter candidates for highly hydrophobic metabolites. In the process of shoot branching, the importance of auxin was already discussed. Also is to consider the newly identified plant hormone strigolactones derived from carotenoids [45,47]. Several genes involved in their biosynthesis have been identified but knowledge of both the biosynthesis pathway and the transport of strigolactones and their intermediates is still incomplete [53]. How some highly hydrophobic intermediates such as carotenoid-derived compound can be transported within the cytoplasm of the cell or on more long distance? The MATE family proteins identified are good candidates for such a function and need now to be considered further.

## Conclusion

Beyond the detection of QTLs for growth and leaf traits, this study explored further the genomic resources as the genome sequence and its annotation to identify candidate genes for the traits under study. For sylleptic branches, candidate genes identified by enrichment tools analysis of genes inside QTL confidence intervals were promising. Nevertheless, the improvement of poplar genome annotation and the reduction of QTL confidence intervals might help to refine this strategy of candidate gene discovery.

## Methods

### Plant material

Poplar material consisted of a cloned 336 F1 progeny from an interspecific cross between the female *Populus deltoides* (Bartr. Ex Marsh.) ‘73028-62’ from Illinois and the male *P. trichocarpa* (Torr. and Gray) ’101-74’ from Washington State. Several pollinations were conducted in 1990, 1995 and 1996 by INRA (Orléans, France) to produce this F1 progeny. Cuttings were produced from stoolbeds of the same age.

### Experimental design and trait measurement

The field trial was established in April 2003 from 25 cm- homogenous hardwood cuttings planted at an initial spacing of 0.75 m x 2 m, accommodating a plant density of 6670 trees per ha. The experimental trial, located in Central France (Ardon, 47°49’41''N, 1°54’39''E, 110 m), consisted in 6 randomized complete blocks where each F1 genotype and each parent was represented by one replicate. To reduce the border effects, a double border row was planted around the plantation. The plantation management included irrigation and the use of insecticides and fungicides as needed throughout two growing seasons.

As productivity traits, circumference and stem height were measured at the end of the first (winter 2003–2004) and second (winter 2004–2005) growing seasons as described in [54]; measured variables were named Circum1, Circum2 and Height1 and Heigth2 respectively. Total number of sylleptic branches (Syllep1) was counted at the end of the first growing season. Growth increment in height and circumference during the second growing season were calculated as: deltaH = Height2–Height1 and deltaC = Circum2–Circum1.

For leaf traits, on September 2^nd^, 2003, one fully illuminated mature leaf was collected on each tree; this leaf presented the largest width along the main stem (Foliar Index between 15 and 17; see [27] for details). Six calibrated discs of lamina (2 cm^2^) were cut from this leaf; leaf discs were then dried at 50 °C during 48 °C and weighed, and specific leaf area (SLA, cm^2^ g^−1^) was computed. Leaf discs were ground to fine powder for analysis of leaf carbon isotope composition (δ^13^C), carbon (C_M_) and nitrogen (N_M_) contents. All analyses were performed at the technical platform of functional ecology at the INRA-Nancy. One-milligram subsamples of ground material were enclosed in tin capsules and combusted. The CO_2_ produced by combustion was purified and its ^13^CO_2_/^12^CO_2_ ratio was analysed by a continuous flux isotope ratio mass spectrometer (IRMS Delta S, Finnigan MAT, Bremen, Germany) with a precision over measurements of ± 0.14 ‰. The discrimination between atmospheric CO_2_ (δ_air_ assumed to be close to −8 ‰) and plant material (δ_plant_) was calculated as Δ = (δ_air_ - δ_plant_) / (1 + (δ_plant_ / 1000)) according to [55]. C_M_ and N_M_ were expressed on a dry weight basis (mg.g^-1^ DW).

### Statistical analysis

For each variable, assumptions on residual distributions of the linear models were checked with the Shapiro–Wilk statistic. All statistical tests were considered significant at *P* ≤ 0.05. For adjustment of individual data to block effect, the following model was used: *Y*_ij_ = *μ* + *B*_i_ + *ε*_ij_ where *μ* is the general mean and *B*_i_ is the effect of block i considered as fixed. *B*_i_ was calculated as the difference between the general mean of the whole family and the mean of block i (*B*_i_ = *Y* – *Y*’_i_).

To evaluate the genetic variation, the following model was used: *Y’*_ij_ = *μ* + *G*_j_ + *ε*_ij_, where *G*_j_ is the effect of genotype j considered as random. Genetic and residual variance components (*σ*_G_^2^ and *σ*^2^_*ε*_) were calculated by equating observed mean squares to expected mean squares and solving the resulting equations according to the Henderson III procedure [56,57]. The coefficient of genetic variation (CV_G_) was estimated as σ_G_ /Mean_Family_. Broad-sense heritabilities were estimated on a genotypic basis, *H*^*2*^_Genotype_ = *σ*_G_^2^/(*σ*_G_^2^ + (*σ*_ε_^2^/*n*_j_)), where *n*_j_ is the average number of replicates per genotype, and on an individual basis, *H*^*2*^_Individual_ = *σ*_G_^2^/(*σ*_G_^2^ + *σ*_ε_^2^) [58]. *H*^*2*^_Genotype_ is reported to give information about the precision of estimated genetic values when genotypic means were used as phenotypic predictors. *H*^*2*^_Individual_ can be considered as a reference value, calculated for one individual, and more easily comparable to literature values. The standard errors of broad-sense heritability were calculated as described previously [59].

Proportion of transgressive segregation was expressed as the percentage of superiority of hybrids over the mean of the two parents: (Mean_Family_-Mean_Parents_)/Mean_Parents_) x 100 [60].

### Genotyping and map construction

Genotyping was conducted using RFLP, STS, RAPD, and microsatellite markers in a first subset of 90 genotypes. Microsatellite and AFLP genotyping was then extended to 253 supplementary genotypes. Marker data were taken from Jorge et al. [61] and 110 new SSR markers were tested. RFLP and STS genotyping was conducted as described previously [62]. The letter “P” followed by a 3- to 4-digit number (*e.g.* P1273) refers to the RFLP markers. RAPD genotyping was performed according to [63]. RAPD markers were called using the Operon kit primer name followed by the molecular weight of the polymorphic band (*e.g.* M02-1150). AFLP genotyping was performed as described in [61]. AFLP markers were named after the code of the *Eco*RI/*Mse*I combination from the kit followed by the band ranking on the gel (e.g. E5M5-7).

The SSR primers came from two different sources: (1) the International *Populus* Genome Consortium, SSR named "PMGC" (http://www.ornl.gov/sci/ipgc/ssr_resource.htm), "ORPM" [64] and "WPMS" [65] and (2) SSR named "ai", "bi" and "bu" developed from public *Populus* spp. EST databases (see Additional file 9 for details).

Different labelling techniques were successively used for SSR genotyping: primers were labelled with γ-^33^P]ATP, with forward fluorochrome-labelled primer or the M13 tailing strategy [66]. These first two were described elsewhere [61]. The third one follow such procedure: PCR reactions were performed in a total volume of 10 μl containing 10 mM Tris–HCl, pH 8.3; 50 mM KCl; 1.5 mM MgCl_2_; 200 μM each of dATP, dCTP, dGTP and dTTP; 0.2 unit Taq Polymerase (Invitrogen); 5 pmoles reverse primer; 0.5 pmoles M13-tailed forward primer; 5 pmoles M13-labelled primer; and 40 ng DNA. PCR were conducted with 42 cycles of a 30-s denaturation at 94°C, 30-s annealing at 55°C or 52°C and 30-s extension at 72°C. Each forward primer was 5’-tailed with the M13 forward consensus sequence. The M13-tailed forward primers were then used in combination with a standard M13 primer labelled with fluorescent dye (6FAM, HEX or NED) at its 5’-end. The amplicons for each SSR marker were separately produced, diluted and pooled post-PCR by three-color multiplexes (6FAM, HEX, and NED) for polymorphism screening. Microsatellite polymorphisms were visualized using an ABI PRISM 3100 genetic analyzer (PE Applied Biosystems, Foster City, Calif.). SSR allele lengths were recorded by GeneScan and Genotyper or Genemapper softwares.

Markers significantly deviating from Mendelian segregation ratios [1:1] (*i.e. P*_*>χ2*_ < 1%) were eliminated from the linkage analysis. Computations were made with Mapmaker version 3.0b [67] and were based on the pseudo-testcross strategy that led to the construction of two parental maps [68]. Steps for the framework map construction were the same than [61]. Briefly, LOD threshold of 3.0 and a recombination fraction of 0.3 were used for grouping markers. Kosambi map function was used to calculate genetic distances. The best order within each linkage group was found using the following succession of commands: ‘order’, ‘compare’, ‘try’ and ‘ripple’ (likelihood difference between the first and second best order = 2.0). Genome coverage of framework genetic maps were calculated using Hulbert et al. [69] method modified by Chakravarti et al. ( [70]; method 3).

### QTL analysis

QTLs were determined using MultiQTL 2.5 (http://www.multiqtl.com/; MultiQTL Ltd, Institute of Evolution, Haifa University, Haifa, Israel). The option ‘marker restoration’ was used to reduce the effect of missing information. The Kosambi mapping function was chosen for recalculation of maps on genotypic data. Single trait analysis was performed using a combination of interval mapping approach and multiple interval mapping. The entire genome was first scanned using the one QTL model and then using the two-linked QTL model. Permutation tests (1000 runs), comparing hypotheses H_1_ (there is one QTL in the chromosome), and H_0_ (no QTL in the chromosome) were run to obtain chromosome-wise statistical significance. In a second step, the genome was scanned for QTLs assuming a two-linked QTL model. For chromosomes for which a single QTL was already detected, permutation tests (1000 runs) were run to compare the hypotheses H_2_ (two-linked QTLs in the chromosome) versus H_1_. Subsequently, when *p*_(H2vsH1)_ < 0.05, permutations were run to compare H_2_ vs. H_0_. A two-linked QTL model was only accepted, if one of the two confidence intervals were coinciding with the peaks of the single QTL model. To speed up calculation time, permutations for the two-linked QTL models were only conducted with 1000 runs when the *p-*value was <0.1 after 100 runs. In a last step, multiple interval mapping was performed including all the significant IM QTLs (single and 2 linked QTLs), and threshold was 0.05. For the remaining significant QTLs, permutations were run per chromosome, using thresholds (0.05) per chromosome. We further computed an adjusted Type I error rate at the chromosome level (α_chr_) using an Type I error rate at whole genome level of 0.05 and the formula: α_chr_ = 1 – {1 – [1 – (1 – α_g_)^1/*M*^}^*m*^ , where *M* is the total number of markers used for the QTL detection on each map and *m* the number of markers in the linkage group carrying the QTL [71]. Bootstrap analysis was performed to estimate the 95% confidence intervals. The total variance explained by all the QTLs for a same trait was estimated by the multiQTL model MIM for each parental map separately. Genetic maps were drawn using Mapchart [72].

### Map and QTL projection on the genome

SSR markers and gene markers position on the *P. trichocarpa* Nisqually-1 genome (http://www.phytozome.net/poplar.php; version 2 of the *Populus* genome assembly) were determined using blast algorithms and primers and probe sequences. An “averaged” ratio of base pair per cM (R) was calculated for each linkage group (LG) and each parental map using linear regression of physical distance (y) on genetic distance (x) with y = 0 when x = 0. This R ratio and positions of the nearest flanking markers were used to calculate physical start (y_Q1_) and physical stop (y_Q2_) positions of QTL confidence intervals following the formulas:

$${\text{y}}_{\text{Q}1}={\text{y}}_{\text{M}1}-\left[\left|{\text{x}}_{\text{M}1}-{\text{x}}_{\text{Q}1}\right|\text{× R}\right]$$

$${\text{y}}_{\text{Q}2}={\text{y}}_{\text{M}2}-\left[\left|{\text{x}}_{\text{M}2}-{\text{x}}_{\text{Q}2}\right|\text{× R}\right]$$

where x_Q1_ and x_Q2_ are the start and stop genome position of the QTL confidence interval, x_M1_ and x_M2_ are genetic map position of the flanking markers. When only two genome anchoring markers were available for a LG, the ratio R was calculated as the base pair distance between the two markers divided by the genetic distance between these two markers. The R ratio for a given LG was used to calculate the physical length of a QTL confidence interval located in another LG only when the former LG belongs to the same chromosome and carries at least one genome anchoring marker. When conflicts existed in the marker order between genetic map and genome sequence, the nearest markers which exhibit an order consistent with the physical order were used. Gene models contained in the physical QTL intervals were retrieved using Biomart tool from Phytozome 8.0 database (annotation version 2.2). When one of the limits (start or stop) of the QTL confidence interval converted in bp fell into a gene model, the gene was included in the QTL interval.

### Gene ontology analysis

Poplar gene models and *Arabidopsis* BLAST results against the TAIR10 version were obtained from Phytozome v8.0 in the version 2.2 of the annotation published in January 16^th^, 2012. Gene Ontology (GO) annotations for the 40,668 poplar gene loci were obtained by mapping the poplar gene loci to the *Arabidopsis* best-hit loci. GO annotations were available for 35,467 poplar gene loci and were mapped to 15,283 *Arabidopsis* loci; 13 and 43 were respectively mitochondrion- and chloroplast-encoded genes and were removed, the reminders are chromosome-encoded.

All enrichment analyses have been realized using the Topology-Weighted (TW, [73]) and model-based gene set analysis (MGSA, [23]) and implemented into Ontologizer web tool [22,74]. The TW method improves the classical enrichment analysis of GO terms by integrating GO graph topology on a global scale and giving to genes annotated with a GO term a weight based on the scores of neighbouring GO terms. On the other hand, MGSA analyses all categories at once by embedding them in a Bayesian network in which gene response is modelled as a function of the activation of biological categories. Probabilistic inference is used to identify the active categories.

## Competing interests

The author(s) declare that they have no competing interests.

## Authors’ contributions

RM collected the field data and interpreted the phenotypic data. JCL performed and interpreted enrichment analysis and helped to draft manuscript. CB analysed quantitative data and helped to draft the manuscript. PFB performed the genotyping of SSR markers and contributed to the map construction. MV selected the *P. deltoides* and *P. trichocarpa* parents and realised the original cross, added RAPD markers in the genetic map and collected the field data. NM collected field data and interpreted phenotypic data. FB supervised the PhD of RM, coordinated the ecophysiological part of the experiment, contributed to the acquisition, the analysis and the interpretation of the phenotypic data, and helped to draft the manuscript. VJ performed and interpreted the QTL mapping, made projection on the genome, collected the genes within QTL intervals, coordinated and wrote the draft manuscript.

## Supplementary Material

Additional file 1 Distributions and relationships between all traits measured. Click here for file

Additional file 2 Summary statistics of the framework genetic maps and status of alignment on the genome. Click here for file

Additional file 3 **Positions of QTLs controlling 11 growth and leaf traits on genetic maps and on the*P. trichocarpa*genome Nisqually-1 v2.2.** Click here for file

Additional file 4 **Position of genome anchoring markers on genetic maps and on genome sequence of *P. trichocarpa *Nisqually-1 v2.2.** Click here for file

Additional file 5 Results of GO term enrichment analysis for all traits. Click here for file

Additional file 6 Detailed annotation of genes with GO terms overrepresented for QTLs controlling number of sylleptic branches (Syllep1). Click here for file

Additional file 7 Comparison of QTLs detected in the present study and in Dillen et al. (2009) and Rae et al. (2008, 2009) works. Click here for file

Additional file 8 Comparison between linkage maps and QTLs on Linkage Group X from the present study and previous studies. Click here for file

Additional file 9 Details of EST – SSR used in the genetic map. Click here for file
