# Supplementary material for: Integrating genome annotation and QTL position to identify candidate genes for productivity, architecture and water-use efficiency in Populus spp
Source: BMC Plant Biol. 2012 Sep 26;12:173. doi: 10.1186/1471-2229-12-173 (PMC3520807; doi:10.1186/1471-2229-12-173)
Supplement: Additional file 1 — Distributions and relationships between all traits measured. [file 1471-2229-12-173-S1.pdf]

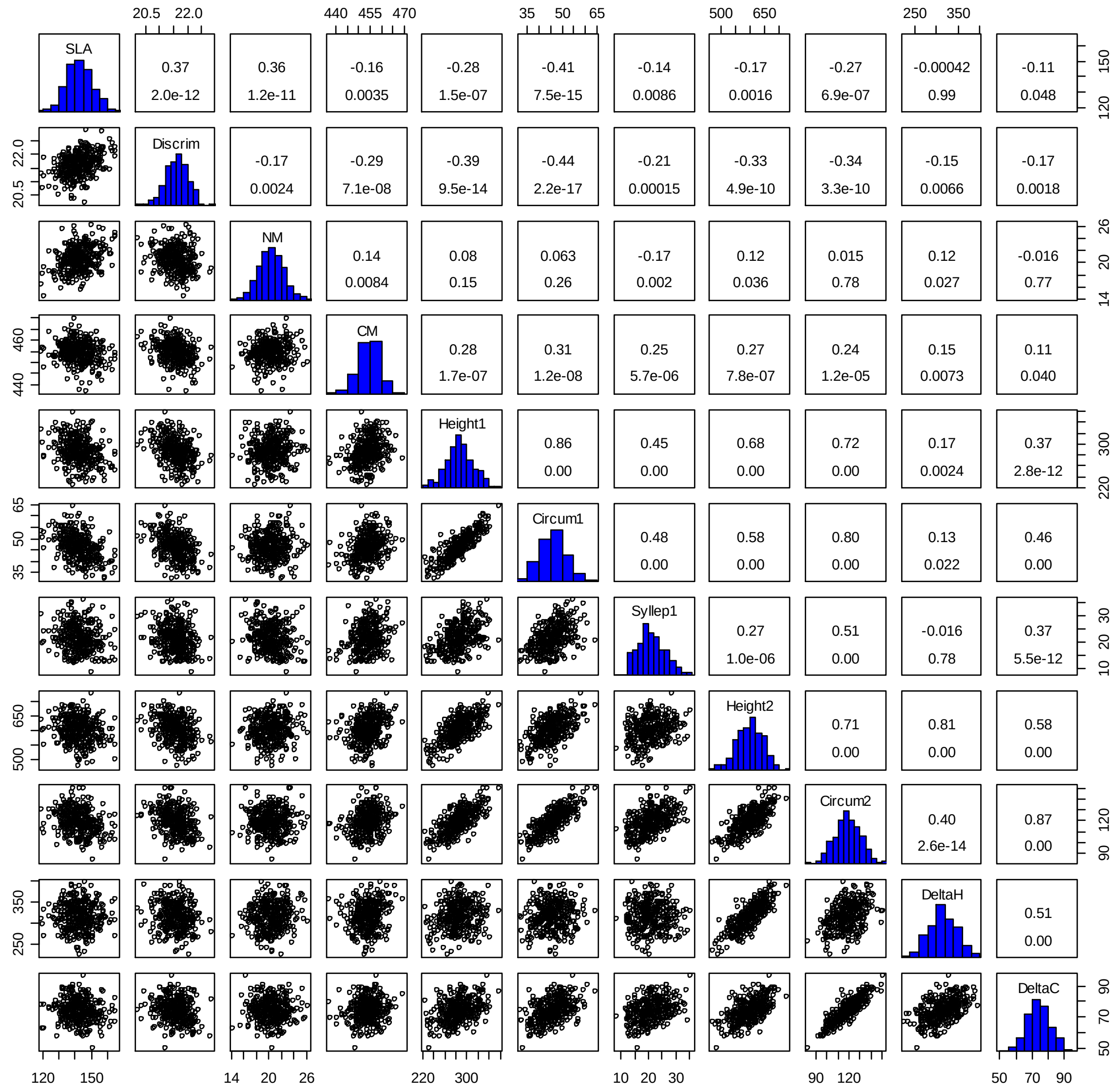

Additional file 1 – Distribution and relationships between all traits measured. Lower panel: scatter plot between all genotypic mean traits; Upper panel: Pearson correlation coefficients and associated *P value*; diagonal: distributions of genotypic means for each. Discrim = Δ.
