# Supplementary material for: Integrating genome annotation and QTL position to identify candidate genes for productivity, architecture and water-use efficiency in Populus spp
Source: BMC Plant Biol. 2012 Sep 26;12:173. doi: 10.1186/1471-2229-12-173 (PMC3520807; doi:10.1186/1471-2229-12-173)
Supplement: Additional file 2 — Summary statistics of the framework genetic maps and status of alignment on the genome. [file 1471-2229-12-173-S2.pdf]

Additional file 2 Summary statistics of the framework genetic maps and status of alignment on the genome.

		Populus deltoides	*P. trichocarpa*
Number of markers		195	208
Number of linkage groups		24	34
Number of genome anchored markers		67	81
Observed map length (cM)		3126.9	3222.8
Mean distance between markers and standard deviation (cM)[a]		18.1±5.3	19.4±6.3
Information on missing data			
	70<N[b]<100	59	82
Number of markers with:	100<N<200	31	14
	200<N<330	105	112
Proportion of missing data		38%	39%

[a] calculated as the average of distances between all adjacent pairs of markers.

[b] N = number of genotyped F1 individuals
