# Supplementary material for: Integrating genome annotation and QTL position to identify candidate genes for productivity, architecture and water-use efficiency in Populus spp
Source: BMC Plant Biol. 2012 Sep 26;12:173. doi: 10.1186/1471-2229-12-173 (PMC3520807; doi:10.1186/1471-2229-12-173)
Supplement: Additional file 3 — Positions of QTLs controlling 11 growth and leaf traits on genetic maps and on theP. trichocarpagenome Nisqually-1 v2.2. [file 1471-2229-12-173-S3.pdf]

Additional file 3 - Positions of QTL controlling 11 growth and leaf traits on parental genetic maps and on the *Populus trichocarpa* genome Nisqually-1 v2.2. (QTL analysis performed using phenotypic data available for 330 F1 progenies)

Parental map	LG	Trait	Position (cM)	95% CI (cM)	LOD	P-value	α_{chr}	Eff.	PVE	Scaffold	R (bp/cM)	Start (bp)	Stop (bp)	CI (bp)	# of genes
P. deltoides	I	C_M	199.3	78.5-291.9	2.421	0.013	0.005	+1.907	0.04	1	95 007	11 009 610	31 500 473	20 490 864	1 835
P. deltoides	I (2-QTL	Circum1	436.4	173.5-436.4	5.921	0.000	0.005	-2.019	0.078	1	95 007	20 035 275	45 228 985	25 193 710	2 280
P. deltoides	model)		144.46	12.8-254.2	5.921		0.005	+2.382	0.078	1	95 007	4 767 650	27 918 709	23 151 060	2 190
P. deltoides	I	N_M	177.36	78.6-276.4	2.833	0.005	0.005	+0.663	0.031	1	95 007	11 019 110	30 027 865	19 008 754	1 677
P. deltoides	I	Height1	434.02	206-436.4	4.096	0.000	0.005	-11.2	0.054	1	95 007	23 123 002	45 228 985	22 105 983	2 069
P. deltoides	II	N_M	54.22	40.7-67.1	9.687	0.000	0.003	+1.576	0.172	2	88 720	4 043 485	4 658 311	614 826	79
P. deltoides	II	Syllep1	60.42	0-159.8	4.866	0.000	0.003	-3.058	0.09	2	88 720	432 581	12 882 655	12 450 074	1 598
P. deltoides	III	C_M	189.58	56.2-232.4	1.995	0.016	0.004	+2.087	0.049	3	85 504	5 201 108	19 590 292	14 389 184	1 665
P. deltoides	IVb	Circum1	55.75	12.2-55.7	2.282	0.007	0.002	+1.987	0.032	4	86 995	-	-	-	-
P. deltoides	IVb	Circum2	55.75	0-55.7	1.341	0.050	0.002	+3.259	0.022	4	86 995	-	-	-	-
P. deltoides	Vb	Circum1	143.97	113-174.5	4.426	0.001	0.004	-3.085	0.076	5	114 195	15 034 861	23 528 371	8 493 510	744
P. deltoides	Vb	Circum2	142.62	71.6-183.1	2.97	0.001	0.004	-5.338	0.059	5	114 195	10 307 188	24 510 448	14 203 260	1 234
P. deltoides	Vb	Syllep1	129.74	98.7-158.5	4.505	0.000	0.004	-2.495	0.06	5	114 195	13 401 873	21 701 251	8 299 379	584
P. deltoides	Vb	SLA	172.09	112.4-208.3	2.614	0.004	0.004	+2.777	0.029	5	114 195	14 475 306	25 802 683	11 327 377	1 072
P. deltoides	Vb	Height1	142.62	47.9-208.3	2.906	0.007	0.004	-11.27	0.055	5	114 195	7 109 728	25 802 683	18 692 955	1 716
P. deltoides	VI	N_M	72.88	32.9-142.2	5.346	0.000	0.003	+1.192	0.098	6	110 354	1 923 237	10 506 877	8 583 640	1 002
P. deltoides	VI	SLA	126.74	93.1-160	3.611	0.006	0.003	+3.942	0.058	6	110 354	8 566 547	12 471 178	3 904 630	366
P. deltoides	VII	C_M	123.1	80.6-161.8	2.767	0.006	0.003	-2.005	0.045	7	92 017	4 153 115	12 118 826	7 965 711	517
P. deltoides	VIII	C_M	52.66	6.6-106.9	1.746	0.034	0.003	+1.51	0.026	8	94 381	475 941	9 849 772	9 373 831	1358
P. deltoides	IXb	deltaC	0	0-55.6	1.993	0.004	0.002	+3.692	0.068	9	110 357	503 968	6 639 798	6 135 830	622
P. deltoides	IXb	SLA	0	0-62	2.226	0.007	0.002	-4.147	0.064	9	110 357	503 968	7 346 083	6 842 114	718
P. deltoides	Xa	Height1	88.75	49.6-119.2	2.906	0.002	0.003	-9.153	0.036	10	113 637	4 866 594	12 489 113	7 622 519	808
P. deltoides	Xa	Height2	99.08	36.6-134	2.213	0.009	0.003	-16.48	0.037	10	113 637	3 389 313	14 170 941	10 781 628	1 075
P. deltoides	Xb	Circum1	27.23	1.7-66.6	4.433	0.000	0.003	+2.79	0.063	10	-	-	-	-	-
P. deltoides	Xb	Syllep1	35.55	4.1-59.6	2.453	0.002	0.003	+1.691	0.028	10	-	-	-	-	-
P. deltoides	XIb	N_M	9.596	0-16.78	2.143	0.000	0.002	-0.62	0.027	11	-	-	-	-	-
P. deltoides	XIa	C_M	18.76	0-53.5	1.544	0.049	0.002	-1.308	0.019	11	-	-	-	-	-
P. deltoides	XIa	SLA	41.42	28.1-58.6	5.307	0.000	0.002	+4.291	0.068	11	-	-	-	-	-
P. deltoides	XIIIb	deltaH	12.74	0-30.1	1.464	0.023	0.003	+15.2	0.057	13	-	-	-	-	-
P. deltoides	XIV	Circum2	74.29	18.3-112.4	2.075	0.019	0.003	-3.586	0.027	14	101 079	5 818 079	12 274 681	6 456 603	819
P. deltoides	XIV	deltaC	70.99	11.7-99.4	3.396	0.001	0.003	-2.998	0.045	14	101 079	5 150 957	10 960 654	5 809 697	786
P. deltoides	XIV	N_M	66.23	1.5-127.4	2.065	0.016	0.003	-0.537	0.02	14	101 079	4 119 952	17 919 311	13 799 359	1 368
P. deltoides	XVI	deltaC	3.778	0-51.7	3.1	0.001	0.003	+3.004	0.045	16	68 722	1	2 798 272	2 798 272	449
P. deltoides	XVI	deltaH	6.682	0-54.6	5.484	0.000	0.003	+19.2	0.091	16	68 722	1	2 997 566	2 997 566	475
P. deltoides	XVI	Height2	11.15	0-84.1	3.007	0.004	0.003	+21.15	0.06	16	68 722	1	5 024 865	5 024 865	694

LG: linkage group; 95% CI: the 95% confidence interval, LOD: the maximum LOD value; P-value: P-value calculated by permutation at chromosome level; α_{chr}: adjusted chromosome P-value for a P-value of 0.05 at genome level; Eff.: the genetic effect; PVE~~, %~~: the proportion of variance explained by the QTL; R: the ration of base pairs per centiMorgan calculated for the respective LG; CI in bp: Confidence intervals defined on the genome in base pair; # of genes: number of genes in the confidence interval. Note that projection was not possible for all QTLs, see Results section. * this interval has been approximated using two markers outside the LG carrying the QTL (see Fig.1).

Additional file 3 (continued)

Parental map	LG	Trait	Position (cM)	95% CI (cM)	LOD	*P*-value	α_{chr}	Eff.	PVE	Scaffold	R (bp/cM)	Start (bp)	Stop (bp)	CI (bp)	# of genes
P. trichocarpa	A	deltaC	27.91	7.3-44.7	1.767	0.014	0.005	-2.59	0.034	-	-	-	-	-	-
P. trichocarpa	Ia	N_M	34.19	0-99.9	2.037	0.019	0.003	-0.626	0.026	1	90 373	1 868 468	10 685 395	8 816 927	1 088
P. trichocarpa	IIb	SLA	3.92	0-36.1	2.633	0.018	0.003	-3.229	0.038	2	60 471	4 841 144	7 024 183	2 183 039	267
P. trichocarpa	IIa	N_M	18.99	0-29.2	2.77	0.002	0.003	+0.862	0.049	2	-	-	-	-	-
P. trichocarpa	IIa	SLA	29.17	12.7-29.2	1.547	0.001	0.003	+2.291	0.019	2	-	-	-	-	-
P. trichocarpa	IV	deltaC	122.42	69.3-198.8	2.948	0.006	0.003	-2.976	0.045	4	86 995	5 749 986	16 998 682	11 248 696	862
P. trichocarpa	IV	deltaH	133.64	3.4-222.3	2.334	0.018	0.003	-13	0.044	4	86 995	783 158	19 043 064	18 259 906	1 658
P. trichocarpa	IV	Height2	107.09	53.2-167.8	2.392	0.010	0.003	-16.56	0.038	4	86 995	4 349 367	14 301 837	9 952 470	730
P. trichocarpa	Vb	Circum2	9.128	0-63.7	2.336	0.013	0.003	+5.011	0.053	5	172 494	8 217 075	18 937 948	10 720 874	760
P. trichocarpa	Vb	deltaC	31.63	0-63.2	4.139	0.000	0.003	+3.003	0.046	5	172 494	8 217 075	18 851 701	10 634 627	757
P. trichocarpa	Vc	Circum2	0	0-13.53	1.417	0.021	0.003	+2.907	0.018	5	-	-	-	-	-
P. trichocarpa	Vc	deltaC	0	0-13.53	2.125	0.003	0.003	+2.141	0.023	5	-	-	-	-	-
P. trichocarpa	Vc	Syllep1	13.53	4.4-13.5	2.477	0.002	0.003	+1.781	0.032	5	-	-	-	-	-
P. trichocarpa	VIb	Δ	40.05	0-44.4	1.48	0.026	0.004	+0.121	0.021	6	*	8 445 158	20 349 732	11 904 574	861
P. trichocarpa	VIb	Height2	1.813	0-22.15	1.332	0.038	0.004	-18.55	0.048	6	*	8 445 158	20 349 732	11 904 574	861
P. trichocarpa	VIc	Circum1	85.81	39.4-85.8	1.498	0.047	0.004	-1.697	0.023	6	91 014	20 085 791	24 307 122	4 221 331	470
P. trichocarpa	VIc	Height1	85.81	42.9-85.8	2.03	0.012	0.004	-8.586	0.032	6	91 014	20 404 340	24 307 122	3 902 782	433
P. trichocarpa	VII	Δ	52.87	12.7-95	2.496	0.001	0.002	-0.147	0.031	7	341 523	1	15 101 417	15 101 417	1 452
P. trichocarpa	VII	Syllep1	88.99	54.1-117.7	2.566	0.008	0.002	+2.321	0.054	7	341 523	12 939 541	15 101 417	2 161 876	263
P. trichocarpa	VII	SLA	53.51	22-90.2	2.658	0.003	0.002	-3.004	0.033	7	341 523	1 976 653	15 101 417	13 124 764	1 125
P. trichocarpa	VIIIa	Circum1	45.4	24.6-60.4	1.765	0.015	0.003	-1.853	0.028	8	62 829	1 720 513	3 969 793	2 249 280	335
P. trichocarpa	VIIIb	deltaC	69.29	11.5-114.6	2.241	0.013	0.003	+3.107	0.049	8	62 175	7 646 308	14 066 021	6 419 713	814
P. trichocarpa	IX	Circum2	57.93	0-131.1	2.077	0.018	0.002	-3.771	0.03	9	56 085	1	10 210 256	10 210 256	1 202
P. trichocarpa	IX	deltaC	57.93	7.9-143	2.146	0.01	0.002	-2.274	0.026	9	56 085	1	10 877 668	10 877 668	1 322
P. trichocarpa	X	deltaH	106.51	46.1-150.8	2.88	0.005	0.002	-10.52	0.029	10	25 222	10 700 239	13 340 985	2 640 746	330
P. trichocarpa	X	Δ	20.5	0-105.7	2.094	0.013	0.002	+0.226	0.072	10	25 222	9 537 504	12 203 472	2 665 968	341
P. trichocarpa	XIb	C_M	0	0-7.31	1.279	0.027	0.003	-1.296	0.018	11	-	-	-	-	-
P. trichocarpa	XII	Circum2	12.58	0-48.2	3.418	0.000	0.001	-4.969	0.052	12	-	-	-	-	-
P. trichocarpa	XII	deltaC	13.65	0-40.9	3.643	0.000	0.001	-3.073	0.048	12	-	-	-	-	-
P. trichocarpa	XII	deltaH	33.24	11.4-57.6	6.299	0.000	0.001	-16.97	0.075	12	-	-	-	-	-
P. trichocarpa	XII	Height2	36.96	13.2-55.4	3.942	0.000	0.001	-19.28	0.052	12	-	-	-	-	-
P. trichocarpa	XIII	deltaH	0.304	0-58.5	3.367	0.001	0.002	+11.53	0.035	13	77 857	569 219	3 510 054	2 940 835	393
P. trichocarpa	XIII	SLA	123.97	115.6-138.9	5.705	0.000	0.002	+5.066	0.093	13	77 857	7 589 841	10 460 837	2 870 996	147
P. trichocarpa	XIII	Height2	0	0-81	2.133	0.012	0.002	+12.82	0.023	13	77 857	569 219	4 895 989	4 326 770	522
P. trichocarpa	XIV	Height1	35.98	0-101.9	1.866	0.035	0.003	-9.532	0.039	14	53 056	2 700 295	9 248 723	6 548 428	911
P. trichocarpa	XV	N_M	67.99	3.4-141.4	1.764	0.031	0.002	-0.577	0.022	15	51 822	3 626 541	9 170 772	5 544 231	395
P. trichocarpa	XVI	Circum2	30.88	0-45	1.434	0.029	0.001	-2.898	0.018	16	9 501	6 790 722	7 218 268	427 546	24
P. trichocarpa	XVI	SLA	30.88	9.1-42.9	1.834	0.015	0.001	+2.429	0.021	16	9 501	6 877 181	7 198 316	321 135	17
P. trichocarpa	XVIIc	deltaH	18.62	0.1-34.8	2.768	0.001	0.002	-17.44	0.079	17	-	-	-	-	-
P. trichocarpa	XVIIc	Height2	18.62	0-34.3	1.788	0.008	0.002	-18.14	0.046	17	-	-	-	-	-
P. trichocarpa	XVIIb	Δ	14.69	8.2-19.6	1.729	0.010	0.002	+0.193	0.052	17	*	788 094	1 696 835	908 741	121
P. trichocarpa	XVIIa	C_M	2.146	0-10.37	1.723	0.006	0.002	-1.692	0.031	17	16 544	788 094	959 656	171562	30
