# Supplementary material for: Integrating genome annotation and QTL position to identify candidate genes for productivity, architecture and water-use efficiency in Populus spp
Source: BMC Plant Biol. 2012 Sep 26;12:173. doi: 10.1186/1471-2229-12-173 (PMC3520807; doi:10.1186/1471-2229-12-173)
Supplement: Additional file 4 — Position of genome anchoring markers on genetic maps and on genome sequence of P. trichocarpa Nisqually-1 v2.2. [file 1471-2229-12-173-S4.pdf]

Additional file 4 - Position of genome anchoring markers on genetic maps and on genome sequence of *P. trichocarpa* Nisqually-1 v2.2.

	Genetic map			Sequence	
**Marker**	**Parent**	**LG**	**Position**	**Scaffold**	**Physical position**[a]
PMGC2550	*P. deltoides*	I	0	scaffold 1	3551560
PMGC2852	*P. deltoides*	I	154	scaffold 1	14548236
bu831219	*P. deltoides*	I	165.5	scaffold 1	17168493
PMGC93	*P. deltoides*	I	170.2	scaffold 1	20634108
PMGC2098	*P. deltoides*	I	264.6	scaffold 1	28906782
PMGC667	*P. deltoides*	II	35.3	scaffold 2	3564397
PMGC2088	*P. deltoides*	II	193.9	scaffold 2	15908007
PMGC2418	*P. deltoides*	II	219.3	scaffold 2	20987627
bu890808	*P. deltoides*	III	0	scaffold 22	40118
bi139327	*P. deltoides*	III	29.7	scaffold 3	1341170
PMGC2879	*P. deltoides*	III	69.8	scaffold 3	6363962
PMGC2611	*P. deltoides*	III	88.5	scaffold 3	8434153
ORPM30	*P. deltoides*	III	138.1	scaffold 3	10738041
PMGC2274	*P. deltoides*	III	207.4	scaffold 3	17452692
ORPM349	*P. deltoides*	IVa	36.3	scaffold 4	530814
PMGC2235	*P. deltoides*	IVb	41.2	scaffold 4	8857455
PMGC639	*P. deltoides*	Va	23.9	scaffold 5	1742818
PMGC576	*P. deltoides*	Vb	96.8	scaffold 5	13184902
PMGC2536	*P. deltoides*	Vb	101.1	scaffold 5	13184902
PMGC2558	*P. deltoides*	Vb	113.2	scaffold 5	20011188
PMGC2839	*P. deltoides*	Vb	172.1	scaffold 5	23254303
PMGC2578	*P. deltoides*	VI	92	scaffold 6	8445158
ORPM190	*P. deltoides*	VI	171.3	scaffold 6	13718178
PMGC2328	*P. deltoides*	VI	207.3	scaffold 6	22164299
PMGC2140	*P. deltoides*	VII	74.9	scaffold 7	3628618
GCPM2741	*P. deltoides*	VII	104.8	scaffold 7	3452654
WPMS17	*P. deltoides*	VII	132.7	scaffold 7	8696000
ORPM312	*P. deltoides*	VII	148.1	scaffold 7	11625105
GCPM2995	*P. deltoides*	VII	158.1	scaffold 7	11778363
PMGC2730	*P. deltoides*	VII	198.9	scaffold 7	14857022
PMGC2607	*P. deltoides*	VIII	33.5	scaffold 8	3014790
PMGC2060	*P. deltoides*	VIII	45.8	scaffold 8	3969793
PMGC61	*P. deltoides*	VIII	74.1	scaffold 8	6435292
PMGC409	*P. deltoides*	VIII	95	scaffold 8	8771675
bi139308	*P. deltoides*	VIII	104.6	scaffold 8	9651572
GCPM2871-1	*P. deltoides*	IXa	4.3	scaffold 9	3720377
PMGC2832	*P. deltoides*	IXa	6.8	scaffold 9	2621937
GCPM588	*P. deltoides*	IXb	0	scaffold 9	9100759
ORPM23	*P. deltoides*	IXb	33.1	scaffold 9	4156785
Win3	*P. deltoides*	Xa	13.6	scaffold 10	775662
PMGC510	*P. deltoides*	Xa	88	scaffold 10	9995391
PMGC2855	*P. deltoides*	Xa	114.6	scaffold 10	11966383
PMGC2786	*P. deltoides*	Xb	34.5	scaffold 10	20146382
PMGC2011	*P. deltoides*	XIa	64.5	scaffold 11	5120414
PMGC2531	*P. deltoides*	XIb	16.8	scaffold 11	17234481
PMGC2737	*P. deltoides*	XII	34.3	scaffold 12	10587866
WPMS05	*P. deltoides*	XII	51.2	scaffold 12	9208512

[a] Physical position is the 5' start of the blast best hit for at least one of the primer.

Additional file 4 (continued)

	Genetic map			Sequence	
Marker	Parent	LG	Position	Scaffold	Physical position[a]
PMGC14	*P. deltoides*	XIIIa	0	scaffold 13	569219
bu810400	*P. deltoides*	XIIIa	5.5	scaffold 13	1023001
ORPM16	*P. deltoides*	XIIIa	42.3	scaffold 30	282451
bu818855	*P. deltoides*	XIIIa	65.9	scaffold 13	4483347
ORPM417	*P. deltoides*	XIIIa	76.6	scaffold 13	11931070
PMGC2658	*P. deltoides*	XIIIa	103.6	scaffold 13	4094900
bu810907	*P. deltoides*	XIV	0	scaffold 14	3968333
ORPM133	*P. deltoides*	XIV	61.1	scaffold 14	7194572
GCPM1377-1	*P. deltoides*	XIV	73.5	scaffold 14	8455977
ORPM193	*P. deltoides*	XIV	77.5	scaffold 14	8780658
CHS	*P. deltoides*	XIV	93.8	scaffold 14	10394612
rDNA	*P. deltoides*	XIV	125.2	scaffold 14	17696937
PMGC2679	*P. deltoides*	XV	31.7	scaffold 15	3265339
PMGC520	*P. deltoides*	XV	68.2	scaffold 15	11798248
ORPM64	*P. deltoides*	XVI	36	scaffold 16	1727740
PMGC433	*P. deltoides*	XVI	70	scaffold 16	3976358
PAL	*P. deltoides*	XVI	116.1	scaffold 16	7223969
PMGC2889	*P. deltoides*	XVII	34.6	scaffold 17	6651152
ORPM263	*P. deltoides*	XIX	32.8	scaffold 19	3116113
GCPM107	*P. deltoides*	XIX	37.9	scaffold 19	419569
Perox1.2	*P. trichocarpa*	Ia	20.5	scaffold 1	3721114
PMGC2499	*P. trichocarpa*	Ia	34.2	scaffold 1	4310925
CCR5	*P. trichocarpa*	Ia	62.5	scaffold 1	2917428
PMGC634	*P. trichocarpa*	Ia	86.4	scaffold 1	9465359
bu867968	*P. trichocarpa*	Ia	121.2	scaffold 1	6882365
bu831219	*P. trichocarpa*	Ib	7.7	scaffold 1	17168493
PMGC93	*P. trichocarpa*	Ib	22.1	scaffold 1	20634108
PMGC2098	*P. trichocarpa*	Ic	0	scaffold 1	28906782
PMGC2084	*P. trichocarpa*	Ic	92.6	scaffold 1	37005906
PMGC2385	*P. trichocarpa*	Ic	176.3	scaffold 1	46095671
PMGC2818	*P. trichocarpa*	IIa	0	scaffold 2	541631
PMGC422	*P. trichocarpa*	IIb	0	scaffold 2	4841144
PMGC684	*P. trichocarpa*	IIb	36.8	scaffold 2	7066513
PMGC223	*P. trichocarpa*	IIc	32.9	scaffold 2	16175440
PMGC2088	*P. trichocarpa*	IIc	46.7	scaffold 2	15908007
ORPM260	*P. trichocarpa*	IIc	60.2	scaffold 2	18844266
PMGC2418	*P. trichocarpa*	IIc	108.2	scaffold 2	20987627
bu890808	*P. trichocarpa*	IIIa	0	scaffold 22	40118
PMGC2277	*P. trichocarpa*	IIIa	14.7	scaffold 22	406512
PMGC2501	*P. trichocarpa*	IIIa	81.4	scaffold 3	8433938
ORPM30	*P. trichocarpa*	IIIa	115	scaffold 3	10738041
PMGC2274	*P. trichocarpa*	IIIb	56	scaffold 3	17452692
PMGC486	*P. trichocarpa*	IIIb	107.6	scaffold 3	19549775
gt18.7	*P. trichocarpa*	IV	0	scaffold 4	487375
PTAG1	*P. trichocarpa*	IV	60.7	scaffold 4	5001829
ORPM127	*P. trichocarpa*	IV	79.9	scaffold 4	6447111
PMGC2881	*P. trichocarpa*	IV	155.1	scaffold 4	14171472

Additional file 4 (continued)

	Genetic map			Sequence	
Marker	Parent	LG	Position	Scaffold	Physical position[a]
ORPM221	*P. trichocarpa*	IV	217.1	scaffold 4	18590690
PMGC2020	*P. trichocarpa*	IV	234.5	scaffold 4	20659627
PMGC639	*P. trichocarpa*	Va	13.6	scaffold 5	1742818
PMGC2536	*P. trichocarpa*	Vb	28.8	scaffold 5	13184902
PMGC2838	*P. trichocarpa*	Vb	44.3	scaffold 5	16936980
PMGC2156	*P. trichocarpa*	Vb	52.1	scaffold 5	16937018
PMGC2839	*P. trichocarpa*	Vc	0	scaffold 5	23254303
PMGC2578	*P. trichocarpa*	Via	68.5	scaffold 6	8445158
ORPM190	*P. trichocarpa*	VIb	44.4	scaffold 6	13718178
PMGC2423	*P. trichocarpa*	VIc	42.3	scaffold 6	20349732
PMGC2328	*P. trichocarpa*	VIc	62.7	scaffold 6	22164299
PMGC2557	*P. trichocarpa*	VIc	85.8	scaffold 6	24307122
GCPM350	*P. trichocarpa*	VII	0	scaffold 7	14508666
GCPM2995	*P. trichocarpa*	VII	50.7	scaffold 7	11778363
PMGC2794	*P. trichocarpa*	VII	60.4	scaffold 7	15091141
PMGC61	*P. trichocarpa*	VIIIa	0	scaffold 8	6435292
PMGC2607	*P. trichocarpa*	VIIIa	45.2	scaffold 8	3014790
PMGC2060	*P. trichocarpa*	VIIIa	60.4	scaffold 8	3969793
PMGC409	*P. trichocarpa*	VIIIb	29.6	scaffold 8	8771675
bi139308	*P. trichocarpa*	VIIIb	43.3	scaffold 8	9651572
ORPM202	*P. trichocarpa*	VIIIb	99.9	scaffold 8	13152048
PMGC2832	*P. trichocarpa*	IX	30.5	scaffold 9	2621937
PMGC2522	*P. trichocarpa*	IX	65.2	scaffold 9	4568105
PMGC2855	*P. trichocarpa*	X	96.3	scaffold 10	11966383
PMGC2571	*P. trichocarpa*	X	106.5	scaffold 10	12223650
SNPT1	*P. trichocarpa*	XIa	18.3	scaffold 11	3525101
PMGC2011	*P. trichocarpa*	XIa	49.6	scaffold 11	5120414
PMGC333	*P. trichocarpa*	XIb	0	scaffold 11	7361508
PMGC2737	*P. trichocarpa*	XII	79	scaffold 12	10587866
PMGC14	*P. trichocarpa*	XIII	0	scaffold 13	569219
bu810400	*P. trichocarpa*	XIII	6.3	scaffold 13	1023001
PMGC2599	*P. trichocarpa*	XIII	66.8	scaffold 13	4156267
bu818855	*P. trichocarpa*	XIII	75.7	scaffold 13	4483347
PMGC649	*P. trichocarpa*	XIII	121.3	scaffold 13	9090554
bi128189	*P. trichocarpa*	XIII	153.8	scaffold 13	13122704
bu810907	*P. trichocarpa*	XIV	23.9	scaffold 14	3968333
PMGC420	*P. trichocarpa*	XIV	58.7	scaffold 14	7275083
PMGC2055	*P. trichocarpa*	XIV	66.6	scaffold 14	7727230
GCPM1292	*P. trichocarpa*	XIV	73.3	scaffold 14	8054865
PMGC571	*P. trichocarpa*	XIV	114.8	scaffold 14	9933145
ai164591	*P. trichocarpa*	XIV	160.8	scaffold 14	11719291
PMGC690	*P. trichocarpa*	XV	27.5	scaffold 15	4875451
PMGC2679	*P. trichocarpa*	XV	68	scaffold 15	3265339
ORPM430	*P. trichocarpa*	XV	164.4	scaffold 15	10362678

Additional file 4 (continued)

	Genetic map			Sequence	
**Marker**	**Parent**	**LG**	**Position**	**Scaffold**	**Physical position**[a]
PMGC2585	*P. trichocarpa*	XV	207.8	scaffold 15	14890771
GCPM598-1	*P. trichocarpa*	XVI	30.9	scaffold 16	7084303
PAL	*P. trichocarpa*	XVI	45.6	scaffold 16	7223969
PMGC2030	*P. trichocarpa*	XVIIa	0	scaffold 17	960152
GCPM3020-1	*P. trichocarpa*	XVIIa	10.4	scaffold 17	788094
PMGC2889	*P. trichocarpa*	XVIIc	45.1	scaffold 17	6651152
PMGC2525	*P. trichocarpa*	XVIII	32.6	scaffold 18	9021159
GCPM79	*P. trichocarpa*	XIX	0	scaffold 19	750932
GCPM2319	*P. trichocarpa*	XIX	11.8	scaffold 19	1558919
GCPM107	*P. trichocarpa*	XIX	37.3	scaffold 19	419569
