# Supplementary material for: Integrating genome annotation and QTL position to identify candidate genes for productivity, architecture and water-use efficiency in Populus spp
Source: BMC Plant Biol. 2012 Sep 26;12:173. doi: 10.1186/1471-2229-12-173 (PMC3520807; doi:10.1186/1471-2229-12-173)
Supplement: Additional file 6 — Detailed annotation of genes with GO terms overrepresented for QTLs controlling number of sylleptic branches (Syllep1). [file 1471-2229-12-173-S6.pdf]

Additional file 6 - Detailed annotation of poplar genes with GO terms overrepresented in QTL confidence intervals for number of sylleptic branches, with corresponding peptides homologs in *Arabidopsis* genome.

**Poplar locus name**	**Arabidopsis transcript name**	**Defline**	**Score**	**Similarity**
POPTR_0002s02690	AT4G31500.1	cytochrome P450, family 83, subfamily B, polypeptide 1	1590	68.1%
POPTR_0002s02700	AT4G31500.1	cytochrome P450, family 83, subfamily B, polypeptide 1	1242	64.5%
POPTR_0002s02730	AT4G31500.1	cytochrome P450, family 83, subfamily B, polypeptide 1	1470	66.5%
POPTR_0002s02740	AT4G31500.1	cytochrome P450, family 83, subfamily B, polypeptide 1	1432	66.6%
POPTR_0002s02770	AT4G31500.1	cytochrome P450, family 83, subfamily B, polypeptide 1	1585	68.5
POPTR_0002s02790	AT4G31500.1	cytochrome P450, family 83, subfamily B, polypeptide 1	1388	63.4%
POPTR_0002s02800	AT4G31500.1	cytochrome P450, family 83, subfamily B, polypeptide 1	1433	65.3
POPTR_0002s09050	AT1G77850.1	auxin response factor 17	1510	56.2%
POPTR_0002s10240	AT5G65380.1	MATE efflux family protein	1873	73.3%
POPTR_0002s10250	AT5G65380.1	MATE efflux family protein	2012	74.8%
POPTR_0002s13340	AT1G47530.1	MATE efflux family protein	788	75.4%
POPTR_0005s17480	AT5G65380.1	MATE efflux family protein	1607	65.4%
POPTR_0007s14390	AT5G65380.1	MATE efflux family protein	2172	80.1%
POPTR_0002s06090	AT3G58850.1	phytochrome rapidly regulated 2 (PAR2)	221	30.9%
POPTR_0005s22330	AT3G58850.1	phytochrome rapidly regulated 2 (PAR2)	211	33.6%

Data obtained from http://www.phytozome.net version 8.0. Peptides homologs are identified through a all-against-all Smith-Waterman protein alignment (see “Help” on Phytozome for further details)
