# Supplementary material for: Integrating genome annotation and QTL position to identify candidate genes for productivity, architecture and water-use efficiency in Populus spp
Source: BMC Plant Biol. 2012 Sep 26;12:173. doi: 10.1186/1471-2229-12-173 (PMC3520807; doi:10.1186/1471-2229-12-173)
Supplement: Additional file 7 — Comparison of QTLs detected in the present study and in Dillen et al. (2009) and Rae et al. (2008, 2009) works. [file 1471-2229-12-173-S7.pdf]

Additional file 7- Comparison of QTL detected in the present study (Monclus et al) and in [5, 8, 9] works. When a QTL has been detected on a linkage group, the cell is coloured in grey.

Traits	Publications	Linkage groups																		
		I	II	III	IV	V	VI	VII	VIII	IX	X	XI	XII	XIII	XIV	XV	XVI	XVII	XVIII	XIX
Height1	Monclus et al	■				■	■				■				■					
	[8]		■	■			■				■			■				■	■	
	[9]			■	■						■			■		■				
	[5]							■			■			■				■		■
Circum1	Monclus et al	■			■	■	■		■		■									
	[8]		■	■							■							■		■
	[9]			■	■	■					■			■						
	[5]							■			■			■						■
Syllep1	Monclus et al		■			■		■			■									
	[8]	■	■		■	■		■	■		■				■		■			
	[9]					■			■		■		■	■	■					
Height2	Monclus et al				■		■				■		■	■			■	■		
	[8]		■	■				■			■							■	■	
	[5]			■				■						■				■		■
Circum2	Monclus et al				■	■				■			■		■		■			
	[8]		■	■							■							■	■	
	[5]							■		■	■			■				■		■
deltaH	Monclus et al				■						■		■	■			■	■		
	[5]			■				■						■				■		
deltaC	Monclus et al				■	■			■	■			■		■		■			
	[5]							■		■	■			■				■		■
SLA	Monclus et al		■			■	■	■		■		■		■			■			
	[9]		■	■					■		■			■			■			
Δ	Monclus et al						■	■			■							■		
	[9]			■		■	■		■		■									
