# Supplementary material for: Integrating genome annotation and QTL position to identify candidate genes for productivity, architecture and water-use efficiency in Populus spp
Source: BMC Plant Biol. 2012 Sep 26;12:173. doi: 10.1186/1471-2229-12-173 (PMC3520807; doi:10.1186/1471-2229-12-173)
Supplement: Additional file 9 — Details of EST – SSR used in the genetic map. [file 1471-2229-12-173-S9.pdf]

Additional file 9 - Details of EST – SSR used in the genetic map.

EST-SSR name	Source [a]	Forward primer	Reverse primer	Ta [b]	Genome localisation	Linkage map localisation
bu831219	BU831219	TTCGGCAGCTCCCATCCAAAAC	GTTCCTCTTCCAAACTGCTTCACC	55°C	Scaffold 1	I
bu867968	BU867968	AGGGTGCAATGGACCATGTC	GGCTCTCTTATTCCACACCG	55°C	Scaffold 1	I
bu890808	BU890808	CCTCCTCAATAATTCAATGGCTGC	GGTAGTAAGAAGTCGAGGTAGG	55°C	Scaffold 3	III
bi139327	BI139327	GCAGAAACCAGCTTCTTGGAC	AATTCCCGACAAGGCTCGAC	55°C	Scaffold 3	III
bi139308	BI139308	ACAATAGACAGCAGGCATGG	GAACGAGAATGTTGGAGGGG	55°C	Scaffold 8	VIII
bi138728	BI138728	TCGCCTCTTATTTGATCGCC	TGCACACATTCCTCTGCCTC	55°C	-	XII
bu810400	BU810400	CAAAAGCTGAAGCTGTGGTAAC	GGCAATGAACATGTCGTCAAAG	55°C	Scaffold 13	XIII
bu818855	BU818855	AAAGGCAAACCCTCCTCCTC	CCTCATCAACCTCTTCCTTGCC	55°C	Scaffold 13	XIII
bi128189	BI128189	GGTTGGTGAACTGATTGAAGAG	GAGACAGATAAACATAGAGAGGGG	55°C	Scaffold 13	XIII
bu810907	BU810907	TTCTTGTTCTCTTCGCAGCGCC	CCAACCCAATGCTTTTCCAGAACC	55°C	Scaffold_14	XIV
ai164591	AI164591	CCACCCAAACCATGCCCTTTATC	GAATCCCCTAAATCGCGCTCAG	55°C	Scaffold_14	XIV

EST-SSRs used for genetic mapping were developed from public *Populus spp*. EST databases. Microsatellites repeats were searched and primers were designed using the GCG package (www.gcg.com, no more developed). PCR and migration conditions were described elsewhere [65]. Forward primers were labeled with fluorochrome 6-FAM or 8-HEX.

[a] Genebank accession number

[b] Temperature of annealing
