# Supplementary material for: Integrating genome annotation and QTL position to identify candidate genes for productivity, architecture and water-use efficiency in Populus spp
Source: BMC Plant Biol. 2012 Sep 26;12:173. doi: 10.1186/1471-2229-12-173 (PMC3520807; doi:10.1186/1471-2229-12-173)

Additional file 8 - Comparison between linkage maps and QTLs on Linkage Group X from the present study and previous studies [5, 8, 9]. All linkage maps were aligned on *P. trichocarpa* Nisqually-1 sequence (black bar, scaffold 10). From the left to the right: *D2* linkage map and QTLs of *P. deltoides* ‘S9-2’ (D×T family, in blue, [5]); *P. deltoides* 73028-62 linkage map and QTLs (in white); *P. trichocarpa* Nisqually-1 physical map with position of genome anchored markers (in black); *P. trichocarpa* 101-74 linkage map and QTLs (in grey); F2 *P. trichocarpa* 93-968 x *P. deltoides* ILL-129 linkage map and QTLs (in green; light green: [8]; dark green: [9]). Marker names are explained in Material and Methods. QTLs were represented by vertical lines or bars indicating start and stop of the confidence intervals. Trait names were explained in Material and Methods.

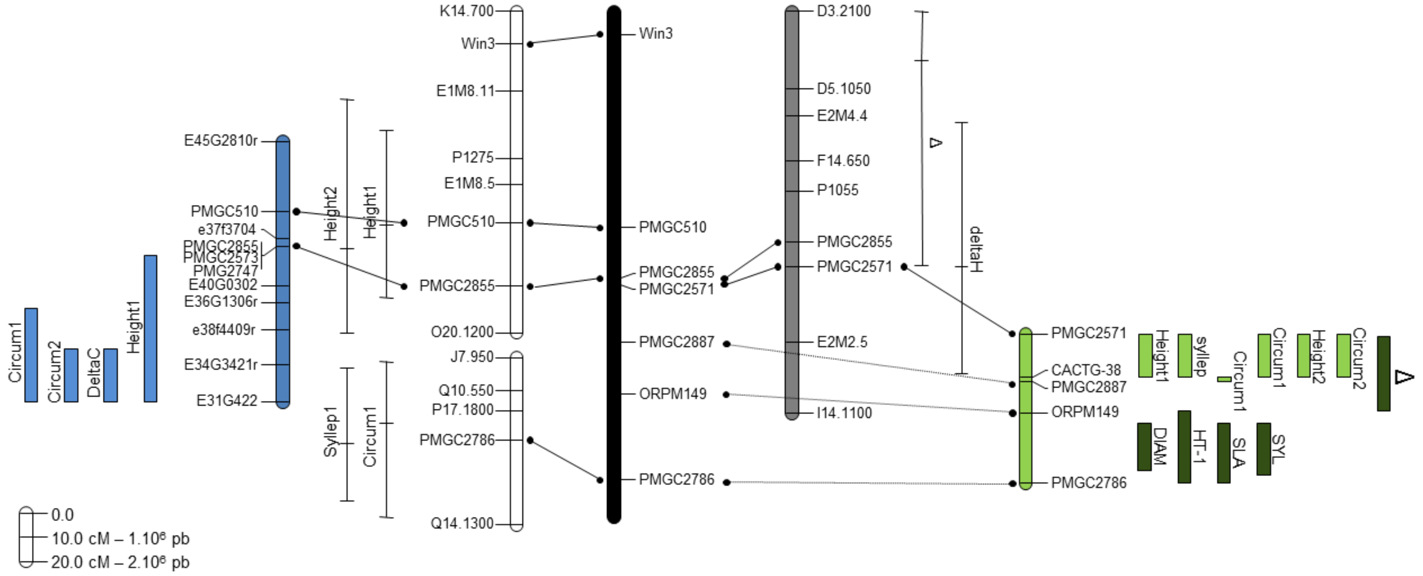

Supplement: Additional file 8 — Comparison between linkage maps and QTLs on Linkage Group X from the present study and previous studies. [file 1471-2229-12-173-S8.pdf]
